# A flow cytometric assay to detect viability and persistence of *Salmonella enterica* subsp. *enterica* serotypes in nuclease-free water at 4 and 25°C

**DOI:** 10.3389/fmicb.2024.1342478

**Published:** 2024-02-16

**Authors:** Anna Williams, Soumana Daddy Gaoh, Alena Savenka, Angel Paredes, Pierre Alusta, Youngbeom Ahn, Dan A. Buzatu

**Affiliations:** ^1^Division of Systems Biology, National Center for Toxicological Research, U.S. Food and Drug Administration, Jefferson, AR, United States; ^2^Division of Microbiology, National Center for Toxicological Research, U.S. Food and Drug Administration, Jefferson, AR, United States; ^3^Office of Scientific Research, Nanotechnology Branch, National Center for Toxicological Research, U.S. Food and Drug Administration, Jefferson, AR, United States

**Keywords:** *Salmonella enterica* subsp. *enterica*, flow cytometry, nuclease-free water, viability and persistence, serotypes

## Abstract

*Salmonella* spp. is one of the most isolated microorganisms reported to be responsible for human foodborne diseases and death. Water constitutes a major reservoir where the *Salmonella* spp. can persist and go undetected when present in low numbers. In this study, we assessed the viability of 12 serotypes of *Salmonella enterica* subsp. *enterica* for 160 days in nuclease-free water at 4 and 25°C using flow cytometry and Tryptic Soy Agar (TSA) plate counts. The results show that all 12 serotypes remain viable after 160 days in distilled water using flow cytometry, whereas traditional plate counts failed to detect ten serotypes incubated at 25°C. Moreover, the findings demonstrate that 4°C constitutes a more favorable environment where *Salmonella* can remain viable for prolonged periods without nutrients. Under such conditions, however, *Salmonella* exhibits a higher susceptibility to all tested antibiotics and benzalkonium chloride (BZK). The pre-enrichment with Universal Pre-enrichment Broth (UP) and 1/10 × Tryptic Soy broth (1/10 × TSB) resuscitated all tested serotypes on TSA plates, nevertheless cell size decreased after 160 days. Furthermore, phenotype microarray (PM) analysis of *S.* Inverness and *S.* Enteritidis combined with principal component analysis (PCA) revealed an inter-individual variability in serotypes with their phenotype characteristics, and the impact of long-term storage at 4 and 25°C for 160 days in nuclease-free water. This study provides an insight to *Salmonella* spp. long-term survivability at different temperatures and highlights the need for powerful tools to detect this microorganism to reduce the risk of disease transmission of foodborne pathogens via nuclease-free water.

## 1 Introduction

Detection of foodborne pathogens is a fundamental requirement for food safety and quality control. In the United States, 31 bacterial pathogens are associated with foodborne illness, with *Salmonella* ranking as the most commonly reported pathogen (Scallan et al., 2011; Adley and Ryan, 2016; Hoelzer et al., 2018; Bell et al., 2021). *Salmonella* is a rod-shaped, Gram-negative facultative anaerobe that belongs to the family *Enterobacteriaceae* (Jajere, 2019). The genus *Salmonella* consist of two major species, *Salmonella enterica* and *Salmonella bongori*, based on differences in their 16S rRNA sequence (Desai et al., 2013). It comprises approximately 2,600 serotypes built on three major antigenic determinants: somatic (O), capsular (K) and flagellar (H) (Brenner et al., 2000). More than 50% of these serotypes (approximately 1,500) belong to *S. enterica* subsp. *enterica*, which accounts for most of the *Salmonella* infections in humans (Desai et al., 2013; Ranieri et al., 2013; Aljahdali et al., 2020; Deaven et al., 2021). Several of these serotypes are characterized by their specific phenotypes, including expression of specific virulence factors and antibiotic resistance profiles, as well as host restriction and adaptation.

According to the Centers for Disease Control and Prevention (CDC), of 250 multistate foodborne outbreaks from 2017 to 2020, *Salmonella* was identified as the predominant cause in 186 cases, followed by *E. coli* in 39 cases and *Shigella* in 25 cases (CDC, 2022). Previous studies identified multiple agricultural practices as sources of contamination that lead to *Salmonella* outbreaks, including using animal manure as fertilizer and contaminated water for irrigation (Steele and Odumeru, 2004; Liu et al., 2018). Water is a well-known reservoir for microbial contamination of fresh produce and a vehicle for pathogen transmission (Levantesi et al., 2012). Fruit and vegetables can become contaminated with pathogens by contact with water. Some of the isolates from these outbreaks were found on produce harvested from farms with irrigation ponds. The PFGE patterns were identical to strains linked to successive *S.* Thompson outbreaks, *S. *Enteritidis outbreaks, and a *S.* Javiana outbreak (Li B. et al., 2014; Liu et al., 2018). A 2021 report described a multistate *Salmonella* outbreak caused by *S. *Typhimurium that was found in packaged salad. The outbreak was traced back to irrigation ponds at the farms (FDA, 2021). The Food Safety Modernization Act (FSMA), which emphasizes the significance of produce safety, established for the first time, science-based minimum standards for the safe growing, harvesting, packing, and holding of fruits and vegetables grown for human consumption. The final rule became effective on January 26, 2016 (FDA, 2016). Previously identified sources of produce contamination, such as agricultural water quality, has been included in the new FSMA standards requirements. Therefore, it is important to understand the viability of *Salmonella* serotypes in water to reduce its pathogenic transmission.

In October 2018, the United States Food and Drug Administration issued a Draft Guidance for Industry called the Standards for the Growing, Harvesting, Packing, and Holding of Produce for Human Consumption. The purpose of this guidance document is to help farms comply with the requirements of the Produce Safety Rule by suggesting practices that reduce foodborne outbreaks (FDA, 2018). There is a strong correlation between *Salmonella* outbreaks and irrigation water. Currently, the standard methods to detect pathogens in food products are mainly based on culture growth. This is a time-consuming process, requiring 2–3 days to obtain positive colonies on selective agar plates.^1^ If further confirmation is required, it will typically take up to one more week to obtain results (Mazumdar et al., 2007). One of the limitations of traditional culture methods for bacterial detection and identification is that they only work for viable, culturable organisms. Under some conditions, bacteria are in a viable but nonculturable (VBNC) state. This is mostly due to environmental stresses, such as temperature, nutrition, and the presence or lack of light. Cells in the VBNC state are characterized by a loss of cultivability on standard media, and are undetectable using conventional plate count techniques (Li L. et al., 2014; Kim et al., 2015; Daddy Gaoh et al., 2022a). Even so, some pathogenic VBNC bacteria such as *Salmonella enterica*, retain their viability and express virulence, which poses a risk to public health. Liao and Shollenberger (2003) reported that *Salmonella* spp. may remain viable for 5 years in sterile water or a phosphate buffered solution in a closed environment at room temperature (25°C). Alternatively, Liu et al. (2018) reported that the presence and persistence of *Salmonella* in water depends on multiple environmental factors, such as temperature, pH, salt, dissolved oxygen concentration, nutrient availability, interaction with other microorganisms, and exposure to UV radiation.

The RAPID-B total plate count (TPC) assay was developed and used to simultaneously count the number of bacterial cells in a sample and distinguish between viable, culturable cells and nonviable, non-culturable (dead and injured) cells in real time (Wilkes et al., 2012; Buzatu et al., 2013). The assay uses two DNA dyes, propidium iodide (PI) and thiazole orange (TO). TO can enter all live and dead cells with varying degrees, bind to DNA, and emit green fluorescence (along the FL1 optical channel). On the other hand, PI is unable to cross an intact cell membrane. It does, however, enter nonviable bacteria with damaged cytoplasmic membranes. PI intercalates with DNA, quenches TO fluorescence, and fluoresces a red signal (FL3 channel) (Wilkes et al., 2012; Buzatu et al., 2013; Williams et al., 2015, 2017; Daddy Gaoh et al., 2022b). In this study, we aim to (1) examine the viability of *Salmonella* serotypes using flow cytometry; (2) compare the survivability of serotypes at different temperatures; (3) observe changes in cell morphology, susceptibility of antibiotics and antiseptics, and (4) look at the kinetics of growth and phenotypes after long-term storage in distilled water.

## 2 Materials and methods

### 2.1 Preparation

#### 2.1.1 Bacterial strains and culture conditions

The *Salmonella enterica* subsp. *enterica* *sero*types used in this study are listed in Table 1. Stock cultures of the serotypes were kept frozen (− 80°C) in 20% glycerol. Initially, each strain was grown on Trypticase Soy Agar plates (TSA; Becton, Dickinson and Company, Sparks, MD) or/and Trypticase Soy Broth (TSB), overnight at 37°C, to ensure pure culture. The cultures grown overnight were used for inoculation.

**Table 1:** *Salmonella enterica* subsp. *enterica* serotype strains and their source.

No	Serotype	Designation and Source	O Antigens	H Antigens (Phase 1)	H Antigens (Phase 2)
1	Bareilly	ATCC 721204	6, 7, 14	y	1, 5
2	Urbana	SAFE Designation 85	30	B	e, n, x
3	Dublin	SAFE Designation 39	1, 9, 12, [Vi]	g, p	-
4	Infantis	ATCC 706717	6, 7, 14	R	1, 5
5	Javiana	ATCC 10721	1, 9, 12	l, z28	1, 5
6	Kentucky	ATCC 744661	8, 20	i	z6
7	Inverness	SAFE Designation 98	38	k	1, 6
8	Cerro	SAFE Designation 100	6, 14, 18	Z_4_, Z_23_	[1, 5]
9	Newport	SAFE Designation 1	6, 8, 20	e, h	1, 2
10	Heidelberg	SAFE Designation 3	1, 4, [5], 12	R	1, 2
11	Enteritidis	SAFE Designation 81	1, 9, 12	[f], g, m, [p]	[1, 7]
12	Typhimurium	SAFE Designation 4	1, 4, [5], 12	i	1, 2

#### 2.1.2 Preparation and storage conditions

For the inoculation of *Salmonella* serotypes in autoclaved nuclease-free water (Ambion™, Austin, TX), six loops of each bacterial strain grown on TSA plates were transferred to 40 mL of autoclaved nuclease-free water in a 50 mL sterile conical tube (Eppendorf, Enfield, CT). The serotypes were adjusted to a density corresponding to 0.1 absorbance at a wavelength of 600 nm (approximate cell density = 10^7^ colony-forming units (CFU/mL)) using the Synergy MX spectrophotometer (BioTek Instruments, Inc. Winooski, VT) and transferred to 2 sterile screw-cap tubes (16 × 125 mm) containing 20 mL of sterile autoclaved nuclease-free water. The inoculated tubes were then stored on a test tube rack, in the dark at room temperature (25°C) and in parallel, on a test tube rack, in a box, to prevent light absorption, at 4°C.

To distinguish between live and dead cells using RAPID-B (RAPID-B is a patented technology – USPTO Patent number 9194868 B2) TPC analysis, different compositions of mixed live and dead cells were assessed. A 1 mL aliquot of *S.* Inverness and *S. *Enteritidis, obtained by scraping 6 loopfuls of cells from TSA plates grown overnight and placing them in two 2 mL microcentrifuge tubes containing distilled water. These were washed 3 times with distilled water by centrifuging 1 min at 15,000 rpm and removing supernatant. Once washed, a 50 μL aliquot of the washed cell solutions was placed into 50 mL conical tube containing 15 mL of distilled water and an optical density of 0.1 at 600 nm was achieved. A 5 mL volume of the cell suspensions was then placed in a thermomixer (Eppendorf, Enfield, CT) for 12 min at 100°C. As little as 2.5 mL from the livestock were mixed with 2.5 mL from the dead/heat treated stock, to create the mixed cell solution. Three sets of dilutions, for the live, mixed, and dead/heat treated cell solutions, were prepared using the original stock solutions (i.e., 100, 50, 0% live cells). To confirm dead cells, the dilutions were grown in duplicate on TSA plates, using a 10 μL volume of inoculum, and incubated overnight at 37°C.

### 2.2 Viability of *Salmonella enterica* subsp. *enterica* serotypes in distilled water

To evaluate the viability of the *Salmonella* serotypes in distilled water, we intentionally inoculated a low level of *Salmonella* serotypes (serial dilutions from 10^7^ to 1 CFU/mL) and monitored bacterial survival for up to 160 days for samples stored at both 4°C and 25°C using flow cytometry and the traditional agar plating method.

#### 2.2.1 RAPID-B total plate count (TPC)

The flow cytometer used in this study was an Apogee Model A50 (Apogee Flow Systems Ltd., Hemel Hempstead, United Kingdom). The RAPID-B TPC method described previously can separate viable from non-viable cells due to the presence of thiazole orange (TO, Sigma-Aldrich) and propidium iodide (PI, Sigma-Aldrich) (Wilkes et al., 2012; Buzatu et al., 2013). To generates green fluorescence (FL1 channel) for live cells, while PI quenches TO and emits red fluorescence (FL3 channel) for dead cells. To assess the survivability of the *Salmonella* serotypes, serial dilutions were treated with TPC reagent and analyzed using flow cytometry. Briefly, to a volume of 667 μL of each dilution in a 2 mL microcentrifuge tube, 333 μL of TPC reagent was added. The mixture was incubated for 5 min at room temperature, under slow shaking and then analyzed on the flow cytometer. The different dilutions of each serotype were analyzed three times, and the event counts per 100 μL were recorded. The flow cytometry results were converted to cells per mL and compared to colony forming units per mL (CFU/mL) obtained from plate counts.

#### 2.2.2 Direct viable plate count method

The viability of each culture was determined by placing 10 μL drop(s) of a bacterial suspension from each tube, in distilled water, onto TSA plates and allowing the plates to incubate at 37°C for 24 h (Ahn et al., 2019). The change in the viable cell counts of each strain during storage was determined at 1 week interval for 160 days. *Salmonella* serotypes were directly inoculated from distilled water cultures. Samples were collected on days 0, 6, 13, 22, 28, 36, 48, 83, 157, and 160. *Salmonella* serotype survival was tested at 4°C and 25°C for 160 days. At each time point, 10 μL of serial dilutions of a specific *Salmonella* serotype was grown on TSA media to enumerate bacterial cells. The plates were incubated at 37°C for 24 h, and the number of colony forming units (CFU) on each plate was counted using the ProtoCOL3 automated plate counter (Synbiosis, Frederick, MD). The number of colonies was used to calculate the concentration of bacteria in each dilution. Plate assays were performed in three to six replicates and the results were averaged. Pictures for each dilution on plates, were also taken, using the ProtoCOL3 and saved for comparison and future reference.

#### 2.2.3 Sensitivity comparison of the flow cytometry assay to traditional agar plate counts

*S.* Inverness and *S. *Enteritidis serial dilutions ranging from 10^1^ to 10^7^ CFU/mL after 160 days in nuclease-free water were examined to compare the sensitivity of the flow cytometry technique to conventional agar plate counts.

### 2.3 Resuscitation with universal pre-enrichment broth (up) and 1/10 × tryptic soy broth (TSB)

To assess the recovery effectiveness of Universal Pre-enrichment Broth (UP) and 1/10 × TSB (Ahn et al., 2014), the *Salmonella* serotypes cultured in distilled water for 160 days at both 4 and 25°C were used. Briefly, a 500 μL volume of culture from each strain was added to 12 individual 2 mL microcentrifuge tubes, containing 1.5 mL each of UP broth and 1/10 × TSB, and incubated at 37°C for 24 h under gentle shaking (110 rpm). Following the 24-h incubation period, 10 μL aliquots for each strain culture using UP and 1/10 × TSB were plated in triplicate, respectively, onto TSA and 1/10 × TSA plates which were subsequently incubated at 37°C for 24 h to determine viability of cells. On the following day, the presence (+) or absence (−) of colonies was noted.

### 2.4 Physiological tests of *Salmonella enterica* subsp. *enterica* serotypes in distilled water

#### 2.4.1 Growth kinetics in tryptic soy broth (TSB) after 160 days

To evaluate the effect of nutrient content on growth kinetics, *S.* Inverness and *S. *Enteritidis cultured in distilled water at initial (T_0_), 30 (T_30_), and 160 days (T_160_), were adjusted to optical density (OD_600_) 0.1 with the Synergy MX spectrophotometer from BioTek Instruments, Inc. (Winooski, VT). A volume of 20-μL of each suspension was transferred to a 96-well plate containing 180 μL per well of 1/10 × TSB. The plate was incubated at 25°C and kinetics of growth was monitored by the Synergy MX spectrophotometer programmed to measure the absorbance at 600 nm every 2 h for 24 h with 10 s of intermittent shaking (Ahn et al., 2014). Growth curves were fitted using DMFit based on the model of Baranyi and Roberts (1994) to calculate the parameters of maximum growth rate (μ_max_) and lag phase duration (λ).^2^

#### 2.4.2 Scanning electron microscopy (SEM)

The effect of long-term storage in distilled water on the morphology of serotypes was examined using a Zeiss Merlin Gemini2 Field Emission Scanning Electron Microscope (Carl Zeiss Company, LLC, White Plains, NY) after 182 days. Two test samples were prepared from 1 mL aliquots of day 182, Culture 7 (*S.* Inverness) incubated at 25°C and at 4°C. The aliquots were placed in two 2 mL sterile microcentrifuge tubes (Costar, Corning Inc., Cambridge, MA). After preparing test samples, control samples were prepared from the 24-h culture of *S.* Inverness by placing 5 loops of cells into a 2 mL microcentrifuge tube containing a 1 mL of autoclaved nuclease-free water. The 3 tubes were centrifuged (Eppendorf 5,425 microcentrifuge) for 10 min at 15,000 rpm and all but ~500 μL of the supernatant was removed. This was done in order to allow the pellet to be dissolved in the remaining supernatant before submission for electron microscopy.

For electron microscopy sample preparation, the samples were washed in nuclease-free water for 5 min (on ice) and spun down using a Legend Micro 21 Desktop Centrifuge, (Thermo Fisher Scientific, Waltham, MA), running at 8,000 × g for 3 min. Once washed, the samples were resuspended and washed again through a graded series of increasing concentrations of ethanol in water consisting of 20, 50, 70, 90% and twice with 100% ethanol for 5 min to dehydrate them. The samples were spun down at 8,000 × g for 3 min and the pellets were resuspended in each concentration of ethanol in the series. Once the samples were dehydrated with ethanol, the samples were pelleted in 100% ethanol and resuspended in hexamethyldisilane (HMDS), (Electron Microscopy Sciences, Cat #16700, Hatfield, PA). HDMS is a chemical with little surface tension that minimizes drying artifacts. It is used to properly dry soft delicate samples for SEM. After washing twice in HMDS, 1–4 μL of resuspended sample were applied to the center of a 5 × 5 mm conductive silicon chip (Ted Pella, Inc., Cat. No. 16008, Redding, CA) attached to center of SEM stubs using 12 mm of conductive double-sided carbon tape (Ted Pella, Inc., Cat. No. 16084–4, Redding, CA). Once air-dried in a chemical hood, the samples (mounted on stubs) were placed in a Denton Desk V sputter coater (Denton Vacuum, Mooretown, NJ) and sputter-coated with gold–palladium for 45 s with tilt and table rotation. Images were recorded using a Zeiss Merlin Gemini2 Field Emission Scanning Electron Microscope (Carl Zeiss Company, LLC, White Plains, NY). Images were recorded at 3.0 kV with the microscope conditions displayed in the data bar in each image, documenting the relevant microscope conditions used.

#### 2.4.3 MICs of antibiotics and benzalkonium chloride (BZK)

The minimum inhibitory concentrations (MIC) of ampicillin, ciprofloxacin, gentamicin, tetracycline and benzalkonium chloride (BZK) for *S.* Inverness and *S. *Enteritidis were verified on a 96-well microtiter plate (Kim et al., 2015). The MICs of antibiotics, as well as the antiseptic BZK were determined by this method: *S.* Inverness and *S. *Enteritidis cells were scraped from the TSA plate cultures grown overnight at 37°C, washed 3 times with nuclease-free water, and placed in a 1 mL volume of distilled water. Serial dilutions of antibiotics and BZK in concentrations ranging from 0.01 to 128 μg/mL, were prepared in 200 μL of Mueller Hinton Broth (MHB, Sigma-Aldrich) medium. The wells were inoculated with 2 × 10^5^ CFU/mL and the plates were incubated for 24 h at 37°C. The MIC of antibiotics and BZK, in which no bacterial growth was observed (OD_600nm_ < 0.05), was determined.

#### 2.4.4 Phenotype microarray (PM) analysis

A selection of Biolog, Inc. (Hayward, CA) Phenotype Microarray’s (PM) were used to assess differences in biochemical and physiological properties in *S.* Inverness and *S. *Enteritidis. There was a total of 6 samples tested: *S.* Inverness control; *S. *Enteritidis control; *S.* Inverness at 25°C; *S.* Inverness at 4°C; *S. *Enteritidis at 25°C; and, *S. *Enteritidis at 4°C. For each serotype, 6 plates (PM1, PM2A, PM3B, PM4A, PM9 and PM10) were inoculated in triplicate with the cell suspension and incubated at 37°C for 24 h. The PMs tested were PM1 and PM2A, which looked at differences in various carbon sources. PM3B looked at differences in nitrogen sources. PM4A looked at differences in phosphorus and sulfur sources. PM9 looked at the differences in osmolytes, and finally PM10 looked at differences in pH. An optical density of as close to 0.1 at 600 nm was achieved for all samples, before following the specified protocol for Phenotype Microarray analysis (Guard et al., 2016). To PM1 and PM2A, 20 mL of the cell suspension (0.1 OD_600 nm_) was added to the dye mix (IF-0 + dye; 1.8 mL of dye mix and 23.2 mL of sterile water into the 125 mL of 1.2 × IF-0) and 100 μL of this suspension was inoculated into each well. To PM3B and PM4A, 200 μL of a 2 M sodium succinate solution was added to the 20 mL cell suspension, having an OD_600 nm_ of 0.1, which was then added to the dye mix, and 100 μL of this suspension was inoculated into each well. To PM9 and PM10, 600 μL of the cell suspension, having an OD_600 nm_ of 0.1, were added to the dye mixture (IF-0 + dye into the 120 mL IF-10 (1,200 dilution)), and 100 μL of this suspension were inoculated into each well.

All of the PM plates were incubated overnight at 37°C and next measured using the Synergy Mx spectrophotometer set absorbance at 590 nm (A_590_) and 750 nm (A_750_). The endpoint reads were performed at 590 nm with subtraction of a 750 nm reference reading (A_590_ - A_750_) which corrects for any background light scatter. To find variations in Biolog plates features, we employed one-way analysis of variance (ANOVA) with a significance level of *p* < 0.05. The ANOVA was carried out using SigmaPlot software ver. 13.0 (Palo Alto, CA). GraphPad Prism 9 ver. 0.10.0 was used to do a Pearson correlation analyses and principal component analysis (PCA).

## 3 Results

### 3.1 Viability assessment of *Salmonella enterica* subsp. *enterica* serotypes in distilled water

#### 3.1.1 Flow cytometry live or dead evaluation

**Figure 1:**
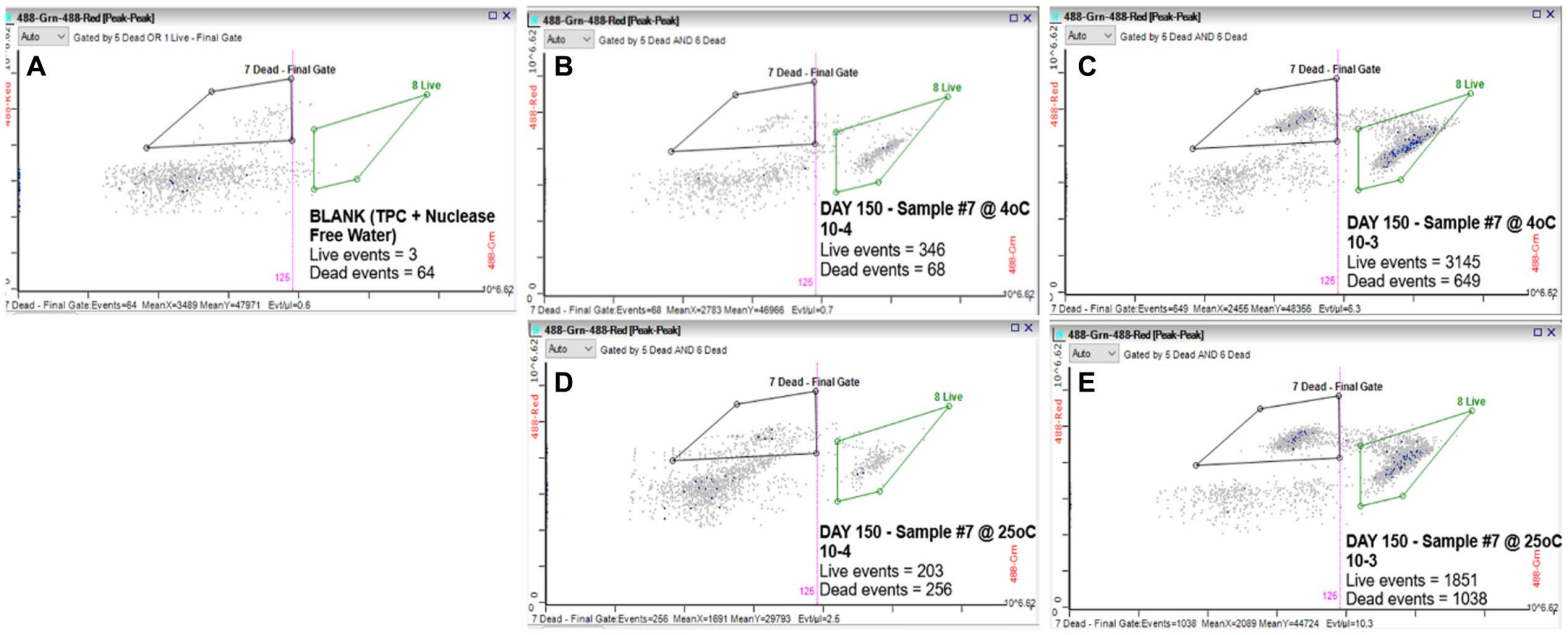
RAPID-B TPC assay flow cytometry cytograms for live/dead cells: **(A)** Blank; **(B)** Day 150 at 4°C with 10 CFU/mL dilution; **(C)** Day 150 at 4°C with 100 CFU/mL dilution; **(D)** Day 150 at 25°C with 10 CFU/mL dilution; and **(E)** Day 150 at 25°C with 100 CFU/mL dilution.

We evaluated the ability of the RAPID-B TPC flow cytometry assay to discern live vs. dead cells by comparing serial dilutions (100 and 10 CFU/mL) of *S.* Inverness suspensions that were stored at 4 and 25°C for 150 days in nuclease-free water. For control purposes, a phosphate buffered saline (PBS) blank (TPC + nuclease-free water) was run on the flow cytometer showing 3 live events and 64 dead events (Figure 1A). The S. Inverness cell counts at 4°*C* were 346/68 (live/dead events) and 3145/649 counts, respectively for the two dilutions (Figures 1B,C). Furthermore, the cell counts at 25°C were 203/256 (live/dead events) and 1851/1038 counts, respectively (Figures 1D,E). The dead events measured by the RAPID-B assay control samples for each dilution series were subtracted from the dead events in each sample, since blank events/counts are not from real bacteria.

#### 3.1.2 Flow cytometry vs. TSA live or dead standard curve

**Figure 2:**
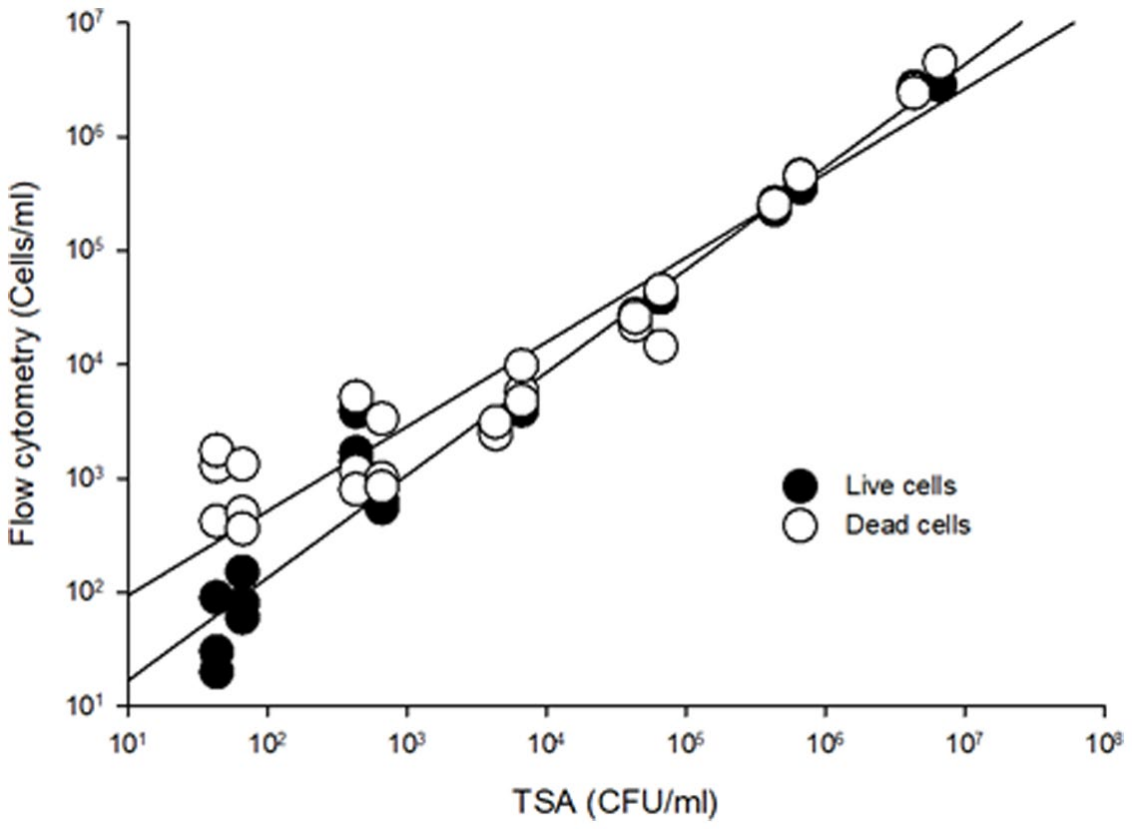
Comparison viable cell counting from TSA plates and RAPID-B TPC flow cytometry assay for *S.* Inverness *and S*. Enteritidis. Live cells (r^2^ = 0.9711, *y* = 2.094*x*^0.902^), dead cells (r^2^ = 0.9779, *y* = 16.932*x*^0.7418^).

To compare the sensitivity of the flow cytometry assay to traditional agar plate counts, serial dilutions ranging from 10^1^ to 10^7^ CFU/mL of *S.* Inverness and *S. *Enteritidis stored in nuclease-free water for 150 days were analyzed (Figure 2). In comparing the RAPID-B TPC assay to plate counts, the live cell count in both assays showed a high degree of linearity (***r***^2^ > 0.95). In addition, the flow cytometer assay was able to quantify live cells down to 10 cells/mL, but the TSA plate limit of detection was 100 CFU/mL. In summary, for live/dead cell counts, the limit of detection for the flow cytometer was 10–100 cells/mL.

#### 3.1.3 Survival of *Salmonella* serotypes in distilled water using flow cytometry and TSA

**Figure 3:**
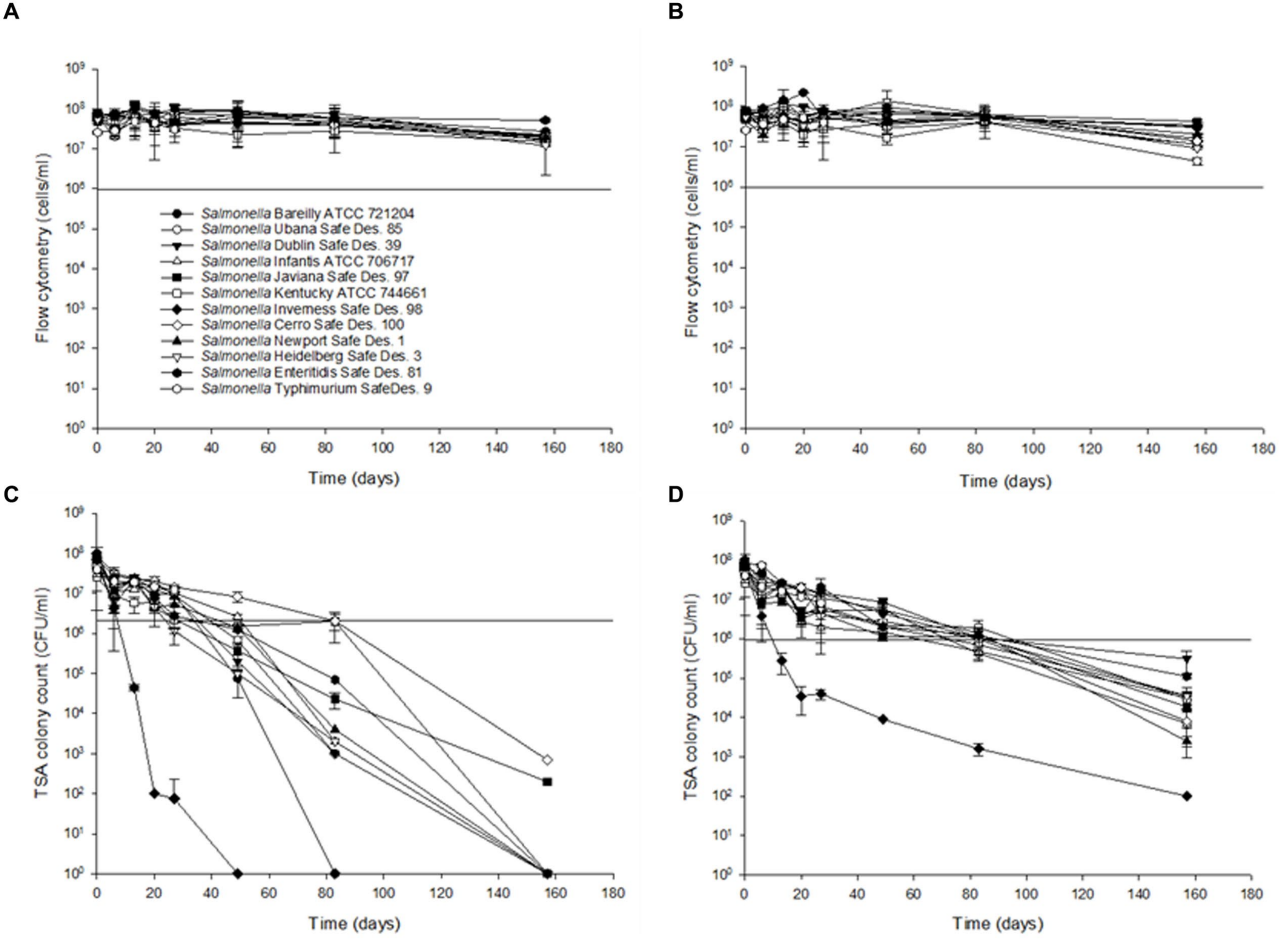
Survival and recovery of Salmonella in distilled water for 160 days at 25°C **(A,C)** and 4°C **(B,D)**. RAPID-B TPC flow cytometry measurements **(A,B)** and TSA plates **(C,D)**. Values are the average of triplicate samples from each of three experiments (*n* = 6). *Y* axis error bars represent standard deviations.

To compare the sensitivity of the flow cytometry assay to traditional agar plate counts, serial dilutions ranging from 10^1^ to 10^7^ CFU/mL of *S.* Inverness and *S. *Enteritidis stored in nuclease-free water for 150 days Using the RAPID-B TPC assay and TSA plates, we determined the persistence of *Salmonella* stored in autoclaved nuclease-free water at 25°C and 4°C over 160 days (Figure 3). Based on flow cytometry analysis, at both 25°C and 4°C, it appears that all 12 of the *Salmonella* serotypes are able to survive in water beyond the 160-day time period (Figures 3A,B). Cell counts were approximately the same at both 25°C and 4*°C* (range 3.3 × 10^7^ ± 1.9 × 10^7^ to 8.0 × 10^7^ ± 6.4 × 10^7^ cells/mL). The cell counts were slightly higher and more variable in *S. *Enteritidis (7.8 × 10^7^ ± 1.7 × 10^7^ and 7.7 × 10^7^ ± 3.4 × 10^7^ cells/mL) than in *S.* Inverness (4.4 × 10^7^ ± 1.4 × 10^7^ and 4.5 × 10^7^ ± 0.8 × 10^7^ cells/mL) at 25°C and 4°C. There were subtle decreases in cell counts for serotype 6 beyond 120 days (from 7.3 × 10^7^ ± 1.5 × 10^7^ to 0.7 × 10^7^ ± 1.9 × 10^7^ cells/mL and 7.3 × 10^7^ ± 3.3 × 10^7^ to 1.2 × 10^7^ ± 0.8 × 10^7^ cells/mL), but the decrease in counts were insignificant.

The survival of *Salmonella enterica* subsp. *enterica* in the experimentally treated distilled water during storage at 4°C and 25°C is shown in Figure 3. To evaluate colony recovery on TSA, 10 μL of serial dilutions 10^7^ to 10^1^ CFU/mL of the 12 *Salmonella* serotypes, cells from distilled water were inoculated onto TSA plates. All of the tested *Salmonella* serotypes ranged from *9.0* ± 1.4 × 10^7^ CFU/mL to *1.2* ± 1.7 × 10^7^ CFU/mL at 25*°C* for 13 days, except Serotype 7 (*6.0* ± 1.4 × 10^7^ CFU/mL to 4.5 ± 0.7 × 10^4^ CFU/mL). After 49 days, *S.* Inverness was not recovered at 25°C. After 83 days, *S.* Bareilly was not recovered at 25°C. After 160 days, with the exception of *S.* Javiana (2.0 ± 1.4 × 10^2^ CFU/mL) and *S.* Typhimurium (7.0 ± 1.4 × 10^2^ CFU/mL), all of the other *Salmonella* serotypes were not recovered at 25°C. However, after 160 days at 4°C, all of the *Salmonella* serotypes were recovered in TSA (10^2^–10^4^ CFU/mL). A noteworthy finding was that *S.* Inverness dramatically decreased after 27 days from 6.0 ± 1.4 × 10^7^ CFU/mL to 4.0 ± 1.1 × 10^4^ CFU/mL, and after 160 days, only 100 CFU/mL were recovered.

#### 3.1.4 Live or dead ratio by RAPID-B total plate count (TPC)

**Figure 4:**
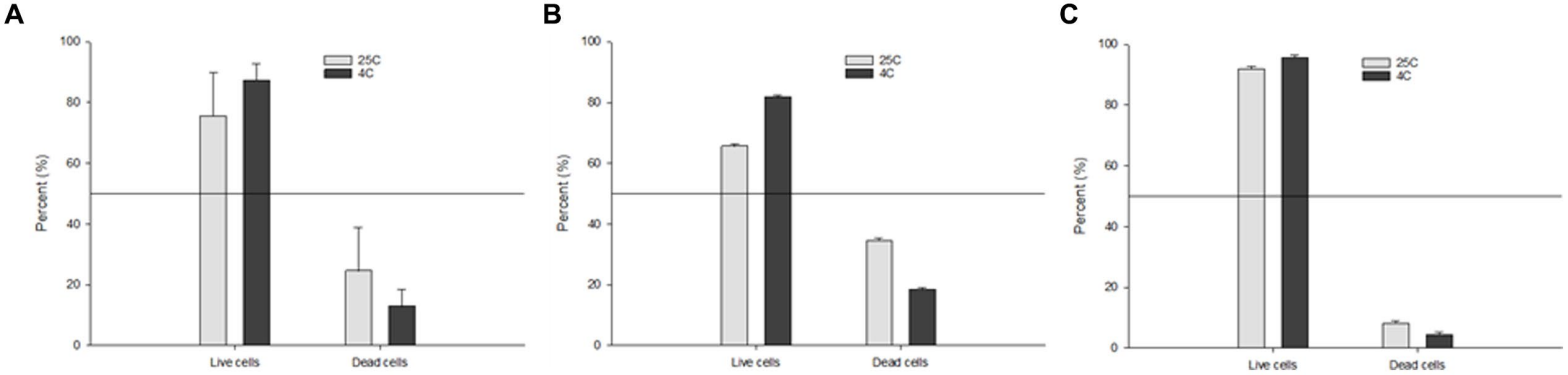
Percentage of the TPC assay live vs. dead cells of: **(A)** all of the tested *Salmonella* serotypes; **(B)** *S.* Inverness; and **(C)** *S. *Enteritidis stored in distilled water at 25°C and 4°C after 160 days.

In order to better understand which storage condition (4°C or 25°C) is more favorable to promote the persistence of *Salmonella* serotypes in water, we assessed the percentage of viable vs. non-viable cells after 160 days using the RAPID-B TPC flow cytometry assay. We also compared the overall percentage of *S.* Inverness with extremely poor TSA plate count recovery and *S. *Enteritidis with strong TSA plate count recovery. The total average percentage of live cells at 25°C and 4°C were 75.5 ± 14.3 and 87.2 ± 5.5 percent, respectively (Figure 4A). By comparison, live cells were slightly higher in *S. *Enteritidis (average 91.9 ± 0.9 and 95.6 ± 0.8) than in *S.* Inverness (average 65.5 ± 0.9 and 81.6 ± 0.8) at 25°C and 4°C. The percentage of live cells was higher at 4°C than 25°C. This is indicative of higher perseverance of *Salmonella* serotypes at lower temperatures.

#### 3.1.5 Resuscitation of nonculturable cells from autoclaved nuclease-free water

**Figure 5:**
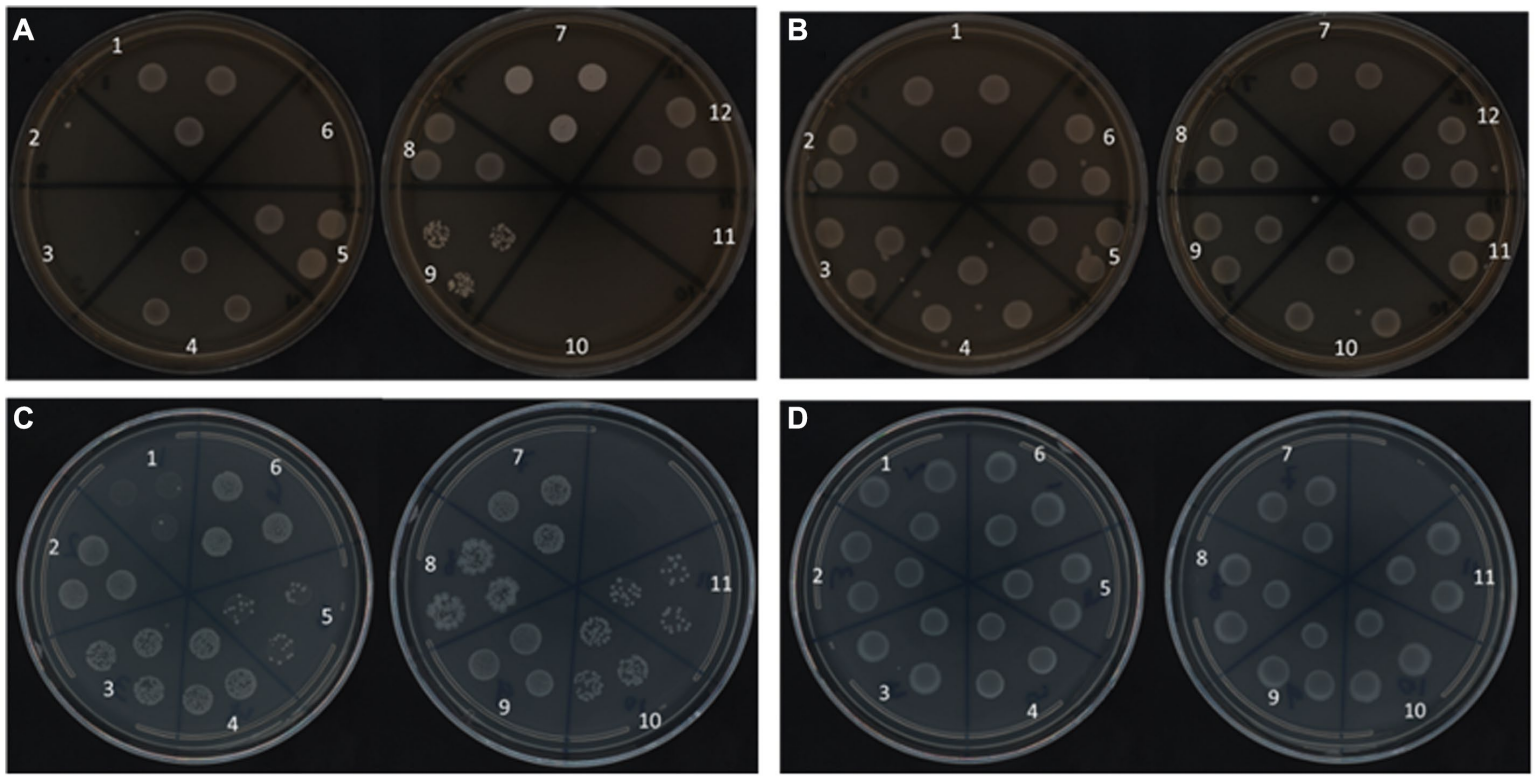
Recovery of *Salmonella* serotypes on TSA plates at 37°C for 24 h from Pre-enrichment with UP **(A,B)** and 1/10 × TSB **(C,D)** at 25°C **(A,C)** and 4°C **(B,D)** after 160 days.

To support the RAPID-B TPC assay flow cytometry finding that all of the *Salmonella* serotypes remain viable in water after 160 days at both temperatures, we used a resuscitation step with UP Broth and 1/10 × TSB enrichment techniques to assess recovery. *Salmonella* serotypes stored at 25°C exhibit a different pattern of recovery based on the treatment. In fact, serotypes *S.* Urbana, *S.* Dublin, *S.* Kentucky, *S.* Heidelberg, and *S. *Enteritidis (2, 3, 6, 10, and 11, respectively) did not recover in Universal Pre-enrichment (UP) Broth (Figure 5A). At 4°C, all 12 serotypes grown overnight in UP broth, and plated onto TSA plates, showed growth (Figure 5B). However, all of the *Salmonella* serotypes in 4°C recovered well on both TSA plates as well as on diluted TSA plates using the enrichment techniques (Figures 5B,D). In contrast, 11 of the 12 serotypes, pre-enriched for 24 h in 1/10 × TSB showed growth on 1/10 × TSA plates at both 25°C and 4°C (Figure 5C). *S. *Typhimurium was not tested on 1/10 × TSB.

### 3.2 Physiological assessment of *Salmonella enterica* subsp. *enterica* serotype after 160 days

#### 3.2.1 Growth kinetics in TSB after 30 and 160 days of *S.* Inverness and *S. *Enteritidis

**Figure 6:**
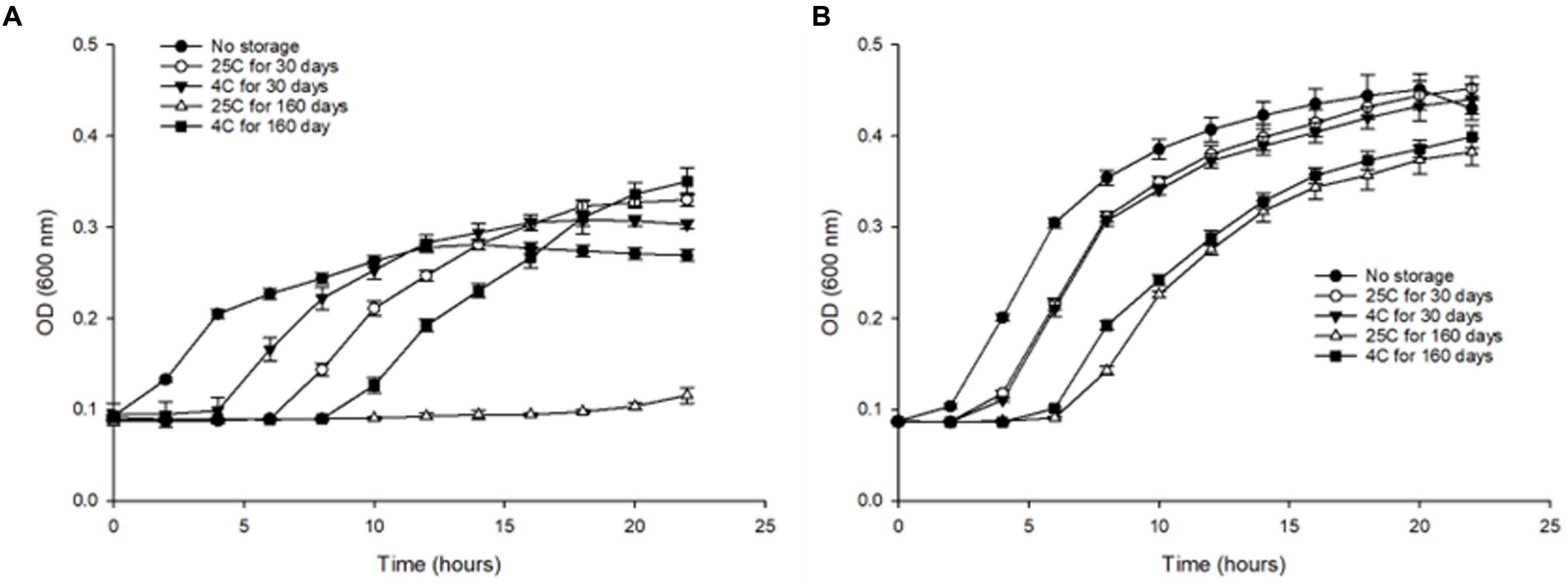
Changes in optical density (at 600 nm) of **(A)** *S.* Inverness and **(B)** *S. *Enteritidis at 37°C for 24 h in TSB media, using 96-well plates.

We evaluated the effect of long-term storage in water at 4°C and 25°C by assessing the growth kinetics, of *S.* Inverness and *S. *Enteritidis after Initial (T_0_), 30 (T_30_), and 160 days (T_160_). Both serotypes were cultured in TSB and inoculated with approximately 10^7^ CFU/mL. For control purposes, fresh cultures of both serotypes were also subjected to the growth kinetic study. Both serotype controls showed noticeably greater growth in TSB. *S.* Inverness and *S. *Enteritidis exhibited the highest growth rate (0.0324 and 0.0403) and the lowest lag phase (1.199 and 1.0562), respectively. Furthermore, compared to *S*. Inverness, *S. *Enteritidis showed greater growth in all conditions tested. However, *S.* Inverness did not grow better in distilled water at 25°C for 30 days (maximum rate, 0.0255; lag, 5.708) and 160 days (maximum rate, 0.00364; lag, 15.591), but instead grew better at 4°C for 30 days (maximum rate, 0.0261; lag, 3.416) and 160 days (maximum rate, 0.0245; lag, 8.359) (Figure 6A). As expected, the samples stored in water for a shorter time (30 days) at both temperatures showed greater growth than those stored in water for a longer duration of time (160 days). However, *S. *Enteritidis at 30 days showed similar growth in distilled water at 25°C (maximum rate, 0.0375; lag, 2.512) and 4°C (maximum rate, 0.0381; lag, 2.731). After 160 days in water, similar results were observed at 25°C (maximum rate, 0.0312; lag, 5.993) and 4°C (maximum rate, 0.0284; lag, 4.717) (Figure 6B). *S.*Inverness did not grow as well in distilled water as *S. *Enteritidis, as can be seen in Figures 6A,B. *S.* Inverness exhibited the most growth at 4°C after being in distilled water for over 160 days, and showed the most growth after being in distilled water for 30 days at 25°C.

#### 3.2.2 Comparison of size and morphology at different temperatures

**Figure 7:**
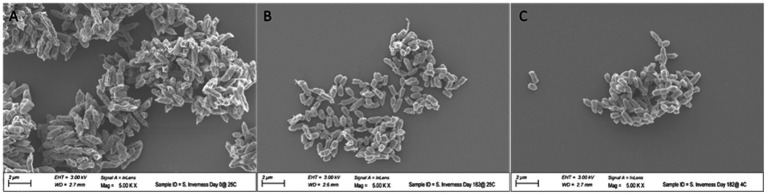
Change in size of *Salmonella* serotype 7 in distilled water at 25°C **(B)** and 4°C **(C)** after 160 days. Control consisted of initial growth at 25°C **(A)**.

To assess the effect of long-term storage at 4°C and 25°C on the morphology of the bacteria, cells of *S.* Inverness, stored for 160 days in distilled water, were collected and subjected to SEM analysis (Figure 7). For control purposes, a fresh culture of the serotype was also submitted for SEM analysis. The control *S.* Inverness (Figure 7A) show a normal rod shape with a smooth surface (approximately 2–3 μm in size). The cells obtained at 25°C (Figure 7B) and 4°C (Figure 7C) after 160 days displayed the same morphology but were under 1 μm in size.

#### 3.2.3 Change of MICs of antibiotics and benzalkonium chloride (BZK) after prolonged incubation in distilled water

The susceptible concentrations of freshly prepared *S.* Inverness and *S. *Enteritidis cells to antibiotics and BZK were compared with the same serotypes that were incubated in distilled water for 160 days at 25°C and 4°C (Table 2). The BZK susceptibility was assessed by broth dilution MIC assays and defined as the cell growth inhibition concentration. For *S.* Inverness, the mean values at initial day (day 0) were 0.5–32 μg/mL at initial day (day 0) and no growth for 160 days at 25°C and 4°C. *S.* Enteritidis was susceptible at similar MIC values on the initial day (0.5–32 μg/mL) and showed no growth after 160 days at 25°C. *S.* Enteritidis showed the same ciprofloxacin susceptible concentration (0.5 μg/mL) after 160 days at 4°C in water as for samples on the initial day samples. However, after 160 days at 4°C in water, they showed high susceptibility of all tested antibiotics as well as of BZK (0.5–16 μg/mL).

**Table 2:** Susceptible concentrations of various antibiotics and BZK for *S.* Inverness and *S.* Enteritidis at 25°C and 4°C for 48 h after 160 days.

Antibiotics/antiseptic	*Salmonella* Inverness Safe Des. 98			*Salmonella* Enteritidis Safe Des. 81		
	Initial	Days 160		Initial	Days 160	
		25°C	4°C		25°C	4°C
Ampicillin	4^a^	ng	ng	4	ng	2
Ciprofloxacin	0.5	ng	ng	0.5	ng	0.5
Gentamicin	2	ng	ng	4	ng	0.5
Tetracycline	4	ng	ng	4	ng	1
Benzalkonium chloride	32	ng	ng	32	ng	16

a concentration (μg/mL).

ng: no growth/recovery in MHB.

#### 3.2.4 Change in metabolic profile for *Salmonella* serotypes using phenotype microarray (PM)

To assess the metabolic profile of the *Salmonella* serotypes under long-term storage in distilled water, we conducted Phenotype Microarray’s (PM) of *S.* Inverness and *S. *Enteritidis using the 96-well plate system. At 24 h, the ΔOD(A_590_ - A_750_) of *S.* Inverness and *S. *Enteritidis ranged from – 0.036 to 1.087. The average of all 4 negative control wells from 24 samples was 0.026 ± 0.02 (A_590_ – A_750_). On the basis of this negative control observation, we considered the ΔOD (A_590_ – A_750_) > 0.05 value as positive. *S. *Enteritidis exhibited greater microbial metabolic activity than *S.* Inverness. There is a statistically significant difference between the control *S.* Inverness and *S. *Enteritidis (initial, fresh culture) and long-term storage *S.* Inverness at 4°C and 25°C (*p* < 0.05) according to the ANOVA and Tukey test. However, no significant difference was observed in *S.* Enteritidis at 4°C and 25°C (*p* > 0.05).

**Figure 8:**
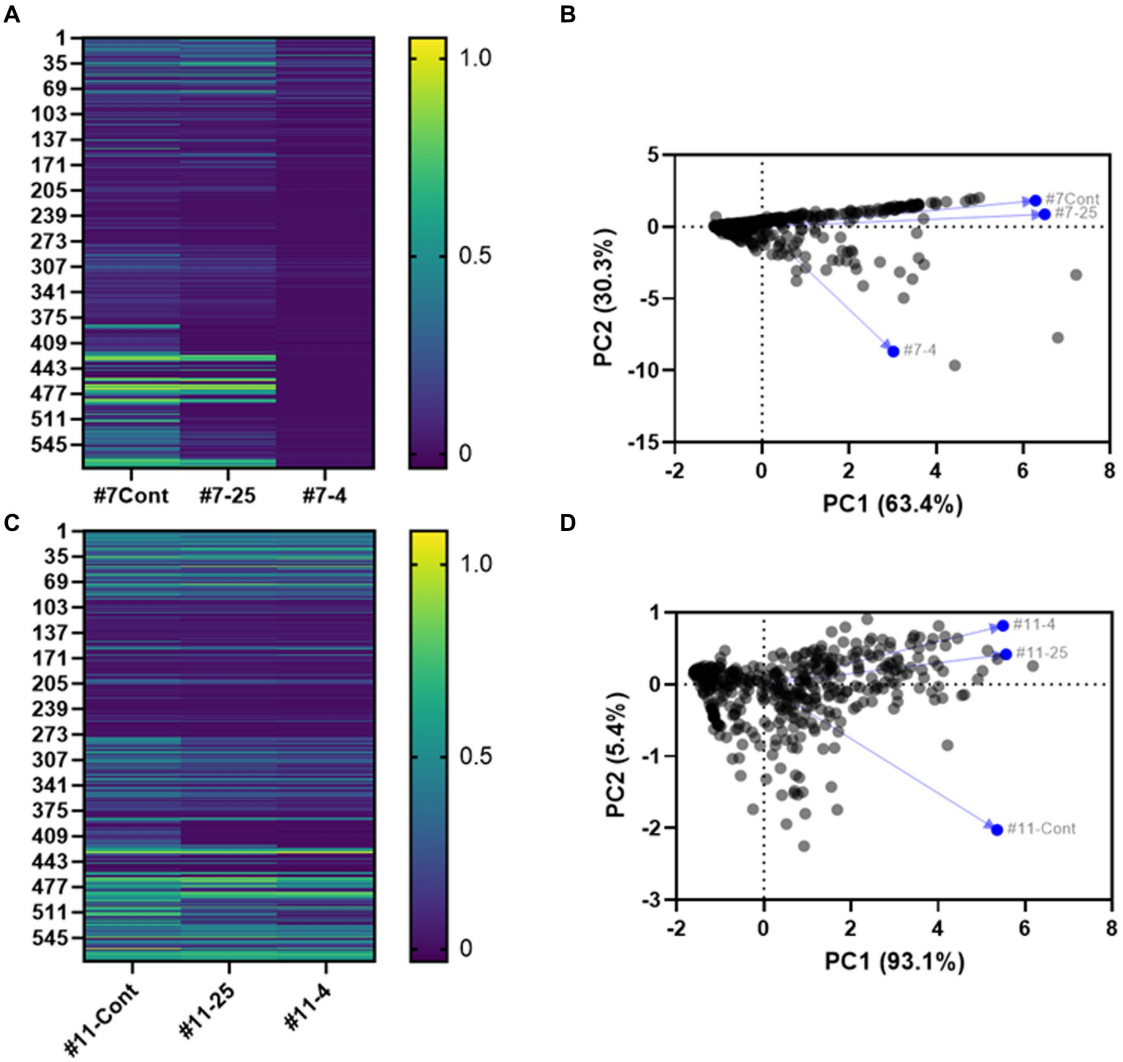
Heatmap **(A,C)** and principal component analysis (PCA) **(B,D)** based on Biolog analysis of 576 compound patterns by *S.* Inverness **(A,B)** and *S. *Enteritidis **(C,D)** at the initial time point (#7- Cont. and #11- Cont.), at 25°C (#7–25 and #11–25) and 4°C (#7–4, and #11–4) for the initial time point vs. 160 days. PM1 and PM2A: Carbon sources, PM3B: Nitrogen sources, PM4A: Phosphorus and sulfur sources, PM9: Osmolytes, PM10: pH.

The heatmap in Figure 8 shows the patterns and trends in metabolite levels in response to long-term storage at 4°C and 25°C of *S.* Inverness and *S. *Enteritidis samples (Figures 8A,C; Supplementary Table S1). The metabolic profile for *S.* Inverness (on the initial day) shows D-glucuronic acid (PM1 B5) and D-glucosamine (PM2A, E5) were the most utilized carbon sources. However, the main carbon sources used at 25°C were thymidine (PM1 C12) and D-tagatose (PM2A, D6), while at 4°C, they were D-glucose-6-phosphate (PM1 C1) and melibionic acid (PM2A, F3). Alternatively, *S. *Enteritidis initially used thymidine (PM1 C12) and melibionic acid (PM2A, F3) as major carbon sources, and after storage at 25°C and 4°C, uridine (PM1 D12) and melibionic acid (PM2A, F3).

For *S.* Inverness and *S. *Enteritidis. The amino acid L-cysteine (PM3B, A11) was the most used nitrogen source initially and during long term storage at 25°C and 4°C. In contrast, these serotypes used phosphorus sources differently. Initially, *S.* Inverness used trimetaphosphate (PM4A, A4) as the phosphorus source, and after storage at 25°C, guanosine-3′-monophosphate (PM4A, B9), and at 4°C, D-3-phospho-glyceric acid (PM4A, B7). *S. *Enteritidis used guanosine-3-monophosphate (PM4A, B9) as a phosphorus source initially and at 25°C, and cytidine-2-monophosphate (PM4A, C8) at 4°C.

Similar selection patterns were also seen for osmolyte sources. Initially, *S.* Inverness used sodium nitrate 20 mM (PM9, H2), and after storage at 25°C, ammonium sulfate pH 8 20 mM (PM9, G10), and NaCl 6% + ectoine (PM9, B7) at 4°C. Ethylene glycol 15% (PM9, D11) was used as the osmolyte source initially and at 4°C, and *S. *Enteritidis used ethylene glycol 20% (PM9, D12) at 25°C.

Finally, *S.* Inverness and *S. *Enteritidis showed considerable differences in the Phenotype Microarray (PM10). The pH at the initial time point (T_0_) for *S.* Inverness was 5.0 (PM10, A4). After long-term storage in distilled water with x-α-D-mannoside (PM10, H10), the pH was 5.5 (PM10, A5). These were the respective primary pH values measured at 25°C and 4°C. In contrast, the pH in phenylethylamine (PM10, G8) at the initial time point (T_0_) for *S. *Enteritidis was 9.5. However, at T = 25°C and 4°C, other sources were used at a much lower pH, pH 5.5 (PM10, A5) and pH 6 (PM10, A6).

Principal component analysis (PCA) also showed inter-individual variability in phenotypic characteristics for *S.* Inverness (Figure 8B) and *S. *Enteritidis (Figure 8D) after long-term storage at 25°C and 4°C in nuclease-free water. This reveals that phenotypic characteristics of *S.* Inverness and *S. *Enteritidis could change as a result from long-term storage in water at 25°C and 4°C. Additionally the phenotypic change observed for *S.* Inverness at 4°C was greater than the change at the initial time point and after storage at 25°C. However, for *S. *Enteritidis, there was a noticeable change in phenotypic characteristics due to long-term storage at 25°C and 4°C compared to the initial time point (Figure 8D). And the phenotypic characteristics associated with the initial time point for *S. *Enteritidis changed more than that of *S.* Inverness at both temperatures.

## 4 Discussion

Typical methods for detection, isolation, and identification of *Salmonella* in water require non-selective and selective pre-enrichment in liquid medium, followed by isolation using selective and differential agar plates (Liu et al., 2018). Such methods are labor intense and time-consuming, which can take 4–5 days to complete. Flow cytometry does not require a selective medium or isolation step for detection of bacteria. Using the RAPID-B TPC flow cytometric assay, washed cells were analyzed in less than 10 min which included a 5 min reagent incubation. The assay can show live, or dead cells. This study which consisted of using the flow cytometer and the TPC assay in comparison to traditional growth plates demonstrated that *Salmonella* serotypes are capable of surviving in distilled water beyond a 160-day time period, at both 25°C and 4°C. It’s important to note that bacterial cells are stressed in water, and traditional methods for detection and characterization of bacterial cells are limited. The present study confirms that RAPID-B flow cytometry enables the counting and/or detection in assessing the viability of *S.* serotypes under adverse conditions, such as long-term storage in 100% distilled water at two different temperatures.

Several studies reported that *Pseudomonas fluorescens, P. viridiflava, Erwinia* spp.*, Xanthomonas campestris, Cytophaga johnsonae, Yersinia enterocolitica, Escherichia coli* O157:H7*, Listeria monocytogenes, Burkholderia pseudomallei, Staphylococcus aureus, Campylobacter* spp. and closely related *Arcobacter* spp. can survive in water for several months and in some cases, up to 16 years (Liao and Shollenberger, 2003; Pumpuang et al., 2011; Liu et al., 2018; Bell et al., 2021). Ahn et al. (2014) showed that *Burkholderia cenocepacia* can persist in distilled water for over 40 days. Specifically, *Salmonella* has been reported as being capable of surviving in water for prolonged periods (Liao and Shollenberger, 2003; Kinsella et al., 2006; Kisluk and Yaron, 2012; Liu et al., 2018). *Salmonella enterica* serotype Mbandaka has been shown to survive in sterile water for over 30 weeks (Liao and Shollenberger, 2003). Furthermore, *Salmonella* spp. (14 strains) were recovered after storage in water for at least 5 years at room temperature. In this study, we showed that 12 *Salmonella* serotypes can persist in sterilized nuclease-free water for over 160 days using a RAPID-B flow cytometer combined with the TPC assay. Based on this, the RAPID-B flow cytometric method demonstrated the highest sensitivity for detection of trace levels of *Salmonella* serotypes. It was already demonstrated that this technology exhibits high sensitivity and accuracy by detecting one single viable cell of *Escherichia coli* serotype O157: H7 in a spinach sample, using novel sample preparations (Buzatu et al., 2013; Williams et al., 2015). By applying this sensitive flow cytometry technology, we were able to detect and count live vs. dead bacterial cells in a stressful environment. This method may potentially replace the conventional or traditional plate count method due to the rapid turnaround time, a high degree of sensitivity, and culture-independence. Although dead samples (negative control) need to show better reduction of random particulate matter and noise, novel and patented photobleaching methods have been developed by Wilkes and coworkers (Wilkes et al., 2012; Buzatu et al., 2013) to aid in the reduction of particulates and background noise. Due to time constraints, those methods were not utilized in this study.

*Salmonella enterica* serotypes stored in sterilized nuclease free water could not be grown on traditional plates (the standard direct viable count method). In order to recover the *Salmonella enterica* serotypes after long-term storage in water, another study used pre-enrichment of nuclease-free water samples in broth prior to plating (Kaper et al., 1977). In fact, Ahn et al. (2014) demonstrated that when broth media was used, the growth was about 10-fold more sensitive than any solid medium tested. In this study, no growth was observed on TSA plates for several serotypes after 160 days storage in water at 25°C. Thus, the decision was made to recover *Salmonella* stored in distilled water using Bacteriological Analytical Methods techniques^3^ and oligotrophic media (Ahn et al., 2019). Based on the weight of peptone, UP broth contains more proteose peptone (5 g/L) compared to 1.5 g/L for 1/10 × TSB. Ahn et al. (2014) proposed that 0.1% peptone provided essentially full protection of bacteria and enabled faster doubling time. Samples recovered in oligotrophic medium (1/10 × TSB) resulted in recovery of all serotypes stored for 160 days in sterilized distilled water at 25°C and at 4°C, compared to their recovery in UP broth and TSA. These results are similar to the findings of Ahn et al. (2014, 2019), which demonstrated that *Burkholderia cepacia* complex strains recovered better using diluted TSB. The high concentration of peptone may inhibit or place metabolic stress on nutrient-limited or depleted water populations of *Salmonella* serotypes. In summary, we demonstrated that 10 × diluted TSB constituted a better enrichment media than UP broth for the recovery of *Salmonella* serotypes from distilled water. In addition, the recovery of microorganisms from distilled water samples increased using a pre-enrichment medium.

The data also showed that recovery of serotypes on TSA plates were better for long-term storage in water at 4°C compared to 25°C. The 25°C temperature appeared to have an adverse effect on the survivability of *Salmonella* in nutrient depleted water. This result is in direct agreement with findings that *Salmonella* can survive at lower temperatures in yogurt (Savran et al., 2018) (Szczawinski et al., 2014), in military (REM’s – Meals Ready-to-Eat) low-moisture foods (Flock et al., 2021), and in halva (Kotzekidou, 1998; Osaili et al., 2017). Additionally, the means in lag phase for 160 days storage in water at 4°C (8.359) was shorter than the means for 160 days storage in water at 25°C (15.591) using the model of Baranyi and Roberts (1994). The lag phase will vary depending on the previous storage condition, including temperature and periods of the organism, since the lag phase is the time required for the cell population to adjust to the food environment and begin to grow. One of the effects of temperature on the survival of *Salmonella* spp. during water storage might be explained by the proportion of unsaturated fatty acids (i.e., hexadecenoic and octadecenoic acids) in the cell membrane (Marr and Ingraham, 1962; Osaili et al., 2017). The ratio of unsaturated fatty acids to saturated fatty acids was higher at 43°C than at 10°C (Weber and Marahiel, 2003). Although we did not attempt to analyze fatty acids in this study, unsaturated fatty acids increase membrane fluidity and permeability. And lower temperatures are known to impact bacterial membrane fluidity, lower diffusion rates, lower enzymatic reactions and metabolism, leading to a reduced growth rate (Weber and Marahiel, 2003).

Another possible reason for the survival of *S.* Inverness and *S. *Enteritidis, is that they have the potential to enter the viable but non-culturable (VBNC) state when faced with stressful conditions (Roszak and Colwell, 1987; Caro et al., 1999). *Salmonella* spp. that was in the VBNC state failed to grow on TSA plates, but demonstrated viability using the RAPID-B TPC assay. Confirming this result, the cells were resuscitated using pre-enrichment methods. Morphological and physiological changes have previously been observed in VBNC cells (Highmore et al., 2018). Long-term storage in distilled water showed a slight decrease in cell size for *S.* Inverness. These results are in agreement with the report of Bakhrouf et al. (2008), who suggested that *Salmonella* spp. can survive for longer periods of time under stressful environmental conditions, resulting in many morphological and metabolic modifications. *Salmonella* spp. are facultative anaerobic Gram-negative, rod-shaped bacteria, generally 2–5 μm long by 0.5–1.5 μm wide (Jajere, 2019). Many other studies have documented that, under stressful environmental conditions, *Vibrio* spp. can morphologically and physiologically change to cope with limited exogenous nutrients (Roszak and Colwell, 1987). Therefore, a decrease in bacterial size for the stressful condition, in comparison to no stress, might be a strategy of survival to minimize lower metabolic rates. During starvation conditions, where there is lack of nutrients, bacteria show a general increase in resistance to high osmotic pressure, high and low temperatures (including freezing), oxidative stress, and presence of solvents (Givskov et al., 1994). Gruzdev and colleagues reported that exposure to carbon source starvation was shown to induce protection against polymyxin B and peroxides, as a cross-tolerance (Gruzdev et al., 2011). In this study, the serotypes *S.* Inverness and *S. *Enteritidis that were kept in water for over 160 days were more susceptible to antibiotics and BZK than at the initial time of inoculation. These results are in agreement with previous studies demonstrating MIC values of *B. cenocepacia* exposed after 40 days of storage in water (Kim et al., 2015). *S.* Inverness and *S. *Enteritidis, stored in nutrient-depleted water for a long time, can become vulnerable to antibiotics and antiseptics. To our knowledge, the mechanism defining how long-term storage in nutrient-depleted water affects the antibiotic/antiseptic susceptibility of *Salmonella* in water has not been identified.

The survival of bacteria in nutrient depleted water might depend on physiological state, tolerance of nutrient depleted environments, interactions with other bacteria, and fluctuations in temperature (Ahn et al., 2014). Previously, it has been shown that the Biolog Microbial Identification system would be a convenient method to analyze differences in metabolism between *Salmonella* serotypes (Hayward et al., 2015). *Salmonella* serotypes Derby and Mbandaka showed differences in metabolism, such as D-galactonic acid-g-lactone and D-glucosaminic acid (Hayward et al., 2015). Based on survival in nuclease-free water, data for *S.* Inverness and *S. *Enteritidis suggests that modified bacterial metabolism contributes to their survival in water. At the start of this study, it was hypothesized that there would be differences between the initial state versus long-term storage, including the sources used for metabolism. This study showed that carbon, nitrogen, phosphorus, and sulfur sources were utilized differently, at the initial time point, and after long-term storage in nutrient depleted water. For example, at the initial time point, *S.* Inverness used 2-deoxy-D-ribose as the sole carbon source, but after 160 days, *S.* Inverness did not use the same carbon source. Perhaps, fresh *S.* Inverness cultures metabolize this carbon source better. The metabolic profiles (Biolog plates) used for the phenotype microarrays were unstable and different based on the water storage temperature and time period.

## 5 Conclusion

*Salmonella* serotypes can persist in sterilized nuclease-free water for over 160 days as determined by RAPID-B flow cytometry analysis. Using the RAPID-B TPC assay, the number of *Salmonella* serotypes analyzed decreased after being stored in water at 4°C for 160 days, but was still higher than the same serotypes stored at 25°C. For confirmation, pre-enrichment with UP broth and 1/10 × TSB resuscitated all screened serotypes in TSA. *Salmonella* serotypes showed a reduction in cell size and changed their metabolic profiles to survive for longer periods of time in a low to no nutrient environment. Under stressful environmental conditions, this direct viable cell count as determined by RAPID-B flow cytometry allows enumeration and detection of actively growing cells, as well as dormant and/or dead cells. Further studies will be performed, which will include additional clean up strategies to assist in the elimination of background noise and particulates for flow cytometry analyses, which could greatly enhance the assay performance. In the future, RAPID-B genetic assays will be developed to detect specific *Salmonella* serotypes.

## Data availability statement

The datasets presented in this study can be found in online repositories. The names of the repository/repositories and accession number(s) can be found in the article/Supplementary material.

## Author contributions

AW: Conceptualization, Data curation, Formal analysis, Investigation, Methodology, Writing – original draft, Writing – review & editing. SG: Conceptualization, Data curation, Formal analysis, Investigation, Methodology, Writing – original draft, Writing – review & editing. AS: Data curation, Formal analysis, Writing – review & editing. AP: Data curation, Formal analysis, Writing – review & editing. PA: Data curation, Formal analysis, Writing – review & editing. YA: Conceptualization, Data curation, Formal analysis, Investigation, Methodology, Writing – original draft, Writing – review & editing. DB: Conceptualization, Data curation, Formal analysis, Funding acquisition, Investigation, Project administration, Writing – original draft, Writing – review & editing.

## Footnotes

^1^ https://www.fda.gov/food/laboratory-methods-food/bam-chapter-5-salmonella

^2^ https://browser.combase.cc/DMFit.aspx

^3^ https://www.fda.gov/food/laboratory-methods-food/bam-chapter-5-salmonella
